# Influence of *CYP2C19*, *CYP2D6*, and *ABCB1* Gene Variants and Serum Levels of Escitalopram and Aripiprazole on Treatment-Emergent Sexual Dysfunction: A Canadian Biomarker Integration Network in Depression 1 (CAN-BIND 1) Study

**DOI:** 10.1177/07067437231203433

**Published:** 2023-10-05

**Authors:** Farhana Islam, Leen Magarbeh, Samar S. M. Elsheikh, Stefan Kloiber, Caroline W. Espinola, Venkat Bhat, Benicio N. Frey, Roumen Milev, Claudio N. Soares, Sagar V. Parikh, Franca Placenza, Stefanie Hassel, Valerie H. Taylor, Francesco Leri, Pierre Blier, Rudolf Uher, Faranak Farzan, Raymond W. Lam, Gustavo Turecki, Jane A. Foster, Susan Rotzinger, Sidney H. Kennedy, Daniel J. Müller

**Affiliations:** 1 Campbell Family Mental Health Research Institute, 7978 Centre for Addiction and Mental Health, Toronto, Ontario, Canada; 2 Department of Pharmacology and Toxicology, 7938 University of Toronto, Toronto, Ontario, Canada; 3 Institute of Medical Science, 7938 University of Toronto, Toronto, Ontario, Canada; 4 Department of Psychiatry, 7938 University of Toronto, Toronto, Ontario, Canada; 5 Interventional Psychiatry Program, 10071 St. Michael's Hospital, Toronto, ON, Canada; 6 Department of Psychiatry and Behavioural Neurosciences, 3710 McMaster University, Hamilton, Ontario, Canada; 7 Mood Disorders Program, St. Joseph's Healthcare Hamilton, Hamilton, Ontario, Canada; 8 Department of Psychiatry, 4257 Queen's University, Providence Care, Kingston, Ontario, Canada; 9 Department of Psychiatry, University of Michigan, Ann Arbor, Michigan, USA; 10 Centre for Mental Health, 7989 University Health Network, Toronto, Ontario, Canada; 11 Department of Psychiatry, 2129 University of Calgary, Calgary, Alberta, Canada; 12 Mathison Centre for Mental Health Research and Education, Hotchkiss Brain Institute, 2129 University of Calgary, Calgary, Alberta, Canada; 13 Department of Psychology and Neuroscience, 3653 University of Guelph, Guelph, Ontario, Canada; 14 The Royal Institute of Mental Health Research, Ottawa, Ontario, Canada; 15 Department of Psychiatry, 3688 Dalhousie University, Halifax, Nova Scotia, Canada; 16 Mechatronic Systems Engineering, 1763 Simon Fraser University, Surrey, British Columbia, Canada; 17 Department of Psychiatry, University of British Columbia, Vancouver, British Columbia, Canada; 18 McGill Group for Suicide Studies, 26632 Douglas Mental Health University Institute, McGill University, Verdun, Quebec, Canada; 19 Department of Psychiatry, 10071 St Michael's Hospital, University of Toronto, Toronto, Ontario, Canada; 20 Keenan Research Centre for Biomedical Science, 518773 Li Ka Shing Knowledge Institute, St Michael's Hospital, Toronto, Ontario, Canada; 21 Department of Psychiatry, Psychosomatics and Psychotherapy, University Clinic of Würzburg, Würzburg, Germany

**Keywords:** Antidepressants, sexual dysfunction, *CYP2C19*, *CYP2D6*, *ABCB1*, pharmacogenetics, depression, adverse events, escitalopram, aripiprazole

## Abstract

**Objectives:**

Treatment-emergent sexual dysfunction is frequently reported by individuals with major depressive disorder (MDD) on antidepressants, which negatively impacts treatment adherence and efficacy. We investigated the association of polymorphisms in pharmacokinetic genes encoding cytochrome-P450 drug-metabolizing enzymes, *CYP2C19* and *CYP2D6*, and the transmembrane efflux pump, P-glycoprotein (i.e., *ABCB1*), on treatment-emergent changes in sexual function (SF) and sexual satisfaction (SS) in the Canadian Biomarker Integration Network in Depression 1 (CAN-BIND-1) sample.

**Methods:**

A total of 178 adults with MDD received treatment with escitalopram (ESC) from weeks 0–8 (Phase I). At week 8, nonresponders were augmented with aripiprazole (ARI) (i.e., ESC + ARI, *n* = 91), while responders continued ESC (i.e., ESC-Only, *n* = 80) from weeks 8–16 (Phase II). SF and SS were evaluated using the sex effects (SexFX) scale at weeks 0, 8, and 16. We assessed the primary outcomes, SF and SS change for weeks 0–8 and 8–16, using repeated measures mixed-effects models.

**Results:**

In ESC-Only, *CYP2C19* intermediate metabolizer (IM) + poor metabolizers (PMs) showed treatment-related improvements in sexual arousal, a subdomain of SF, from weeks 8–16, relative to *CYP2C19* normal metabolizers (NMs) who showed a decline, *F*(2,54) = 8.00, *p *< 0.001, *q *= 0.048. Specifically, *CYP2C19* IM + PMs reported less difficulty with having and sustaining vaginal lubrication in females and erection in males, compared to NMs. Furthermore, ESC-Only females with higher concentrations of ESC metabolite, S-desmethylcitalopram (S-DCT), and S-DCT/ESC ratio in serum demonstrated more decline in SF (*r* = −0.42, *p *= 0.004, *q *= 0.034) and SS (*r* = −0.43, *p *= 0.003, *q *= 0.034), respectively, which was not observed in males. ESC-Only females also demonstrated a trend for a correlation between S-DCT and sexual arousal change in the same direction (*r* = −0.39, *p *= 0.009, *q *= 0.052).

**Conclusions:**

*CYP2C19* metabolizer phenotypes may be influencing changes in sexual arousal related to ESC monotherapy. Thus, preemptive genotyping of *CYP2C19* may help to guide selection of treatment that circumvents selective serotonin reuptake inhibitor-related sexual dysfunction thereby improving outcomes for patients. Additionally, further research is warranted to clarify the role of S-DCT in the mechanisms underlying ESC-related changes in SF and SS. This CAN-BIND-1 study was registered on clinicaltrials.gov (Identifier: NCT01655706) on 27 July 2012.

## Abrégé

**Objectifs:**

La dysfonction sexuelle apparue pendant le traitement est fréquemment déclarée par les personnes souffrant de trouble dépressif majeur (TDM) sur les antidépresseurs, qui ont des effets négatifs sur l’observance et l’efficacité du traitement. Nous avons investigué l’association des polymorphismes dans les gènes pharmacocinétiques codant pour les enzymes métabolisant les médicaments du cytochrome-P450, CYP2C19 et CYP2D6, et la pompe d'efflux transmembranaire, la glycoprotéine P (c'est-à-dire ABCB1), sur les modifications de la fonction sexuelle (FS) et de la satisfaction (SS) liées au traitement dans l'échantillon CAN-BIND-1.

**Méthodes:**

178 adultes souffrant de TDM ont reçu un traitement d’escitalopram (ESC) à compter des semaines 0–8 (Phase I). À la semaine 8, les nonrépondants ont été augmentés par aripiprazole (c.-à-d., ESC + ARI, *n* = 91), tandis que les répondants ont continué ESC (c.-à-d., ESC-seulement, *n* = 80) pendant les semaines 8–16 (Phase II). FS et SS ont été évaluées à l’aide de l’échelle SexFX aux semaines 0, 8, et 16. Nous avons évalué les premiers résultats, le changement de FS et SS pour les semaines 0–8 et 8–16, à l’aide des modèles à effets mixtes à mesures répétées.

**Résultats:**

Dans ESC-seulement, les CYP2C19 IM + PM ont montré des améliorations liées au traitement en termes d'excitation sexuelle, un sous-domaine de FS, des semaines 8–16, relativement aux NM du CYP2C19 qui ont montré un déclin, *F*(2,54) = 8,00, *p* < 0,001, *q* = 0.048. Spécifiquement, les CYP2C19 IM + PMs ont rapporté moins de difficulté à avoir et garder une lubrification vaginale chez les femmes et une érection chez les hommes, comparé aux NM. En outre, les femmes à ESC-seulement avec des concentrations plus élevées de métabolite ESC, S- desméthylcitalopram (S-DCT) et le rapport S-DCT/ESC dans le sérum ont démontré plus de déclin dans la FS (*r* = −0,42, *p* = 0,004, *q* = 0,034) et SS (*r* = −0,43, *p* = 0,003, *q* = 0,034), respectivement, ce qui n’a pas été observé chez les hommes. Les femmes ESC-seulement ont aussi démontré une tendance à la corrélation entre S-DCT et un changement d’excitation sexuelle dans la même direction (*r* = −0,39, *p* = 0,009, *q* = 0,052).

**Conclusions:**

Les phénotypes des métaboliseurs du CYP2C19 peuvent influencer les changements d’excitation sexuelle liés à la monothérapie ESC. Ainsi, le génotypage préventif de CYP2C19 peut aider à guider la sélection du traitement qui contourne la dysfonction sexuelle liée à SSRI, ce qui améliore de cette manière les résultats pour les patients. En outre, des recherches supplémentaires sont nécessaires pour clarifier le rôle de S-DCT dans les mécanismes sous-jacents des changements liés à ESC dans la FS et SS.

## Introduction

Impairments in sexual function (SF) and sexual satisfaction (SS) are commonly experienced symptoms of major depressive disorder (MDD). Selective serotonin reuptake inhibitors (SSRIs), used for the treatment of MDD, have variable effectiveness on the management of sexual dysfunction in patients with some individuals reporting improvements in SF and SS with treatment, while others reporting unchanged or worsening of preexisting problems or even emergence of new impairments in SF and SS following the start of treatment.^1^

Disturbances in SF with SSRIs can be attributed to their mechanism of action, which results in elevated levels of synaptic serotonin (5-hydroxytryptamine [5-HT]), a key inhibitory modulator of SF, both centrally and peripherally.^2‐4^ Further, SSRIs have also been shown to activate 5-HT_2A_ and 5-HT_2C_ receptors inhibiting dopaminergic and adrenergic pathways that facilitate sexual motivation and behaviours.^5‐7^ The rates of sexual dysfunction associated with SSRI treatment have been reported to be between ∼25% and ∼80%, with the lower cited rates more likely a function of underreporting by patients in trials that did not specifically assess these side effects.^3,8‐11^ There is also considerable variation in the type and intensity of sexual side effects experienced between patients on SSRIs. Common treatment-emergent sexual side effects include problems with sexual desire (e.g., lack of libido), arousal (e.g., erectile dysfunction or decreased vaginal lubrication), and anorgasmia.^11,12^ Therefore, determining risk factors for treatment-emergent sexual dysfunction, which negatively impacts medication adherence, may improve efficacy for individuals on SSRIs.^13,14^

Genetic polymorphisms underlying interindividual differences in drug pharmacokinetics may be a contributing factor to the observed variability in treatment-emergent sexual dysfunction. Many SSRIs are metabolized by cytochrome P450 (CYP) hepatic enzymes, some of which are polymorphic and show substantial differences in enzymatic capacity. Escitalopram (ESC), an SSRI previously linked with inducing sexual dysfunction in both women and men,^15,16^ is metabolized primarily by CYP2C19, CYP2D6, and CYP3A4.^17^ Allelic variants of the *CYP2C19* and *CYP2D6* genes convey a wide range of enzymatic activity including fully functional, reduced or null function, or having increased activity.^18^ We and others have shown that individuals with reduced or null CYP2C19 and CYP2D6 enzymatic activity, referred to as intermediate metabolizer (IM) and poor metabolizer (PM), respectively, show higher concentrations of ESC in blood compared to individuals with wild-type enzyme function (i.e., normal metabolizers [NMs]), whereas those with increased enzyme activity, known as rapid metabolizers (RMs) or ultrarapid metabolizers (UMs), show relatively lower ESC concentrations.^18‐22^ Hence, interindividual differences in serum concentrations of ESC influenced by *CYP2C19* and *CYP2D6* gene variants may be contributing to variations in the type and intensity of treatment-emergent sexual dysfunctions that patients experience.

Furthermore, the central nervous system concentrations of some SSRIs are regulated by the P-glycoprotein (P-gp) transmembrane efflux transporter located on the blood–brain barrier, which actively transports many endogenous and exogenous compounds against the concentration gradient to keep the levels of these substrates low in the brain. *In vivo* studies have shown that when ATP-binding cassette, subfamily B, member 1 gene (*ABCB1*), which encodes P-gp, was knocked out in mice, increased concentrations of antidepressants that are substrates of this transporter were detected in the brain of mutant mice as compared to controls.^23^ Therefore, variants of *ABCB1* that reduce the activity and expression of P-gp may potentially result in higher brain concentrations of the SSRI substrate, thereby enhancing 5-HT inhibition of SF. Previous studies have suggested that *ABCB1* polymorphisms are associated with response and remission to SSRIs, but their impact on SSRI-linked sexual dysfunction remains to be elucidated.^23‐25^

One approach to reducing sexual dysfunction induced by SSRIs is to use augmentation therapy with an atypical antipsychotic, particularly one that is an agonist at the dopamine D_2_ receptor, which plays a fundamental role in the control of sexual functioning in both males and females.^26^ Aripiprazole (ARI), a partial agonist at the dopamine D_2_ receptor, has been shown to be beneficial in reducing sexual dysfunction induced by antidepressant and antipsychotic agents.^27,28^ It is also one of the first antipsychotics to be approved for use as adjunctive therapy for MDD by the US Food and Drug Administration.^29^ The pharmacokinetics of ARI have previously been shown to be affected by variations in the *CYP2D6* and *ABCB1* genes,^30,31^ but the influence of variations in these genes on the effectiveness of ARI in relieving SSRI-induced sexual dysfunction has not been explored.

In this study, we investigated the effects of variations in the *CYP2C19*, *CYP2D6*, and *ABCB1* genes on treatment-related changes in SF and SS in patients with MDD on ESC monotherapy or ESC with ARI augmentation from the nationwide Canadian Biomarker Integration Network in Depression 1 (CAN-BIND-1) study.^32^ We hypothesized that IM or PM *CYP2C19* and *CYP2D6* metabolizer phenotypes, and *ABCB1* genotypes associated with lower P-gp function or expression,^33^ would show a greater decline in SF and SS from baseline relative to those with normal activity. In patients on adjunctive ARI therapy, we also expected *CYP2D6* metabolizer phenotypes to influence the reversal of any Phase I deteriorations in SF and SS during Phase II following the introduction of ARI. Furthermore, we explored the association between serum levels of ESC, ARI, and their metabolites on treatment-related changes in SF and SS. We expected higher concentrations of ESC and lower concentrations of ARI in serum to be associated with declines in SF and SS.

## Methods

### Participants

The CAN-BIND-1 study (ClinicalTrials.gov identifier: NCT01655706) has been described in detail elsewhere.^32^ Study protocol was approved by the institutional Research Ethics Board at each site. All study participants provided written informed consent. Participants (*N* = 211; age range: 18–61 years; 61.8% female) meeting the criteria for a major depressive episode for a duration of at least 3 months according to the Diagnostic and Statistical Manual for Mental Disorders, Fourth Revision, Text Revision (American Psychiatric Association, 2000) and with a total score of ≥24 on the Montgomery–Åsberg Depression Rating Scale (MADRS) were recruited from 6 Canadian academic health centres. Further inclusion criteria were MDD diagnosis using the Mini International Neuropsychiatric Interview Version 6.1, being psychotropic medication free for ≥5 half-lives at baseline visit, and fluency in English.

### Treatment Protocol

**Figure 1.**
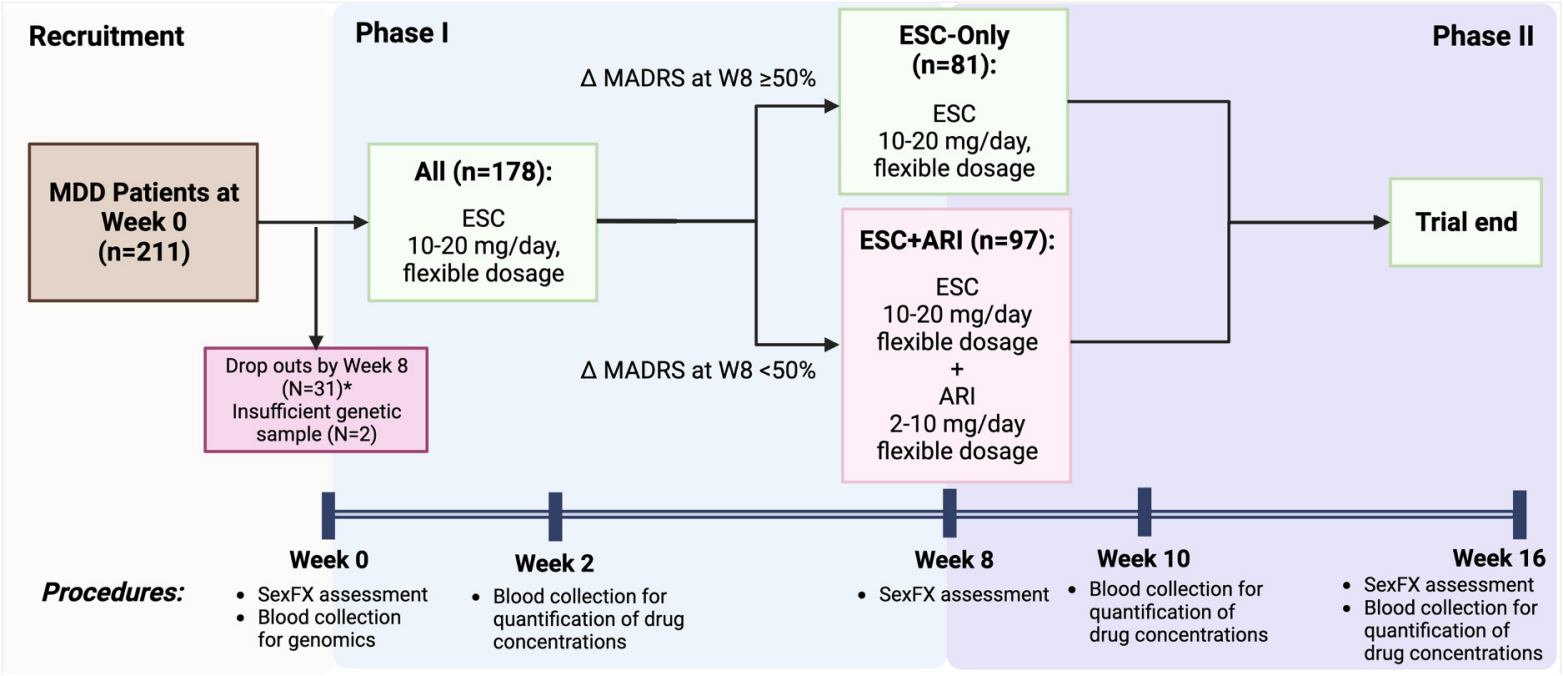
Overview of study protocol.

*Note*. ARI = aripiprazole; ESC = escitalopram; MADRS = Montgomery–Åsberg depression rating scale. *See Kennedy et al.^31^ for a description of the drop-outs.

Following screening and baseline visits, participants commenced treatment with ESC at 10 mg/day, with the option of increased dosages at follow-up visits, to a maximum of 20 mg/day (Figure 1). At week 8, participants with ≥50% or <50% reduction from the baseline MADRS total score were classified as responders and nonresponders, respectively. Week 8 responders continued ESC monotherapy (referred to as ESC-Only), while week 8 nonresponders received adjunctive treatment with ARI at 2–10 mg/day (referred to as ESC + ARI) for an additional 8 weeks. Blood samples were collected on weeks 2, 10, and 16 to measure concentrations of study medications and in week 4 for pharmacogenetic analyses. Collection and measurement of blood samples are described in the Supplemental Methods.

### Measures

SF and SS were assessed using the 13-item clinician-administered sex effects (SexFX) questionnaire at weeks 0, 8, and 16 (Supplemental Table S1). On a 5-point ordered-frequency scale (0–4), SexFX evaluates 3 domains of SF: desire (items 1–4, Cronbach's α = 0.96), arousal (items 5–8, α = 0.60), and orgasm (items 9–11, α =  0.87).^34^ The summation of items 1–11 produces the SF score. A preorgasm score was obtained by combining the desire and arousal domains (α = 0.89). SS is measured by summing items 12 and 13, each scored on a 0–10 scale. Cronbach's alphas for the SF and SS items were 0.92 and 0.85, respectively. This questionnaire is unique from the sexual functioning questionnaire (SFQ) as it measures SS in addition to sexual functioning changes and also has comparable reliability for the latter (Cronbach's alpha of 0.92 for SexFX vs. 0.90 for SFQ).^35,36^

The primary outcomes of the current study are changes in SF and SS for weeks 0–8 and weeks 8–16. Higher scores are associated with better SF or SS.

### Genotyping

A modified version of the FlexiGene DNA Kit (QIAGEN, Hilden, Germany) was used for genomic DNA extraction from whole blood samples. Genotyping for the *CYP2C19*, *CYP2D6*, and *ABCB1* genes was performed using standard TaqMan® assays (Thermo Fisher Scientific, ON, Canada) following the manufacturer's protocol. *CYP2D6* copy number variations (CNVs), including allele deletions and multiplications, were assessed using copy number assays (Thermo Fisher Scientific) and CopyCaller Version 2.1 (Applied Biosystems, Burlington, ON, Canada). Four replicates were performed for the CNV assays. The overall phenotype for *CYP2D6* duplications was determined using the results from the single nucleotide polymorphism (SNP) and CNV assays (e.g., genotype is reported as *1/*3 [xN] if SNP assays revealed *1 and *3 and CNV assay showed more than 2 copies of the *CYP2D6* gene for the same participant). TaqMan® genotyping assay IDs for *CYP2C19* and *CYP2D6* are listed in Supplemental Table S2. Genotyping results were reviewed by two laboratory staff blind to the clinical data.

We translated allelic variants of the *CYP2C19* (*1, *2, *3, and *17) and *CYP2D6* (*1, *2, *3, *4, *5, *6, *9, *10, *17, *29, *36, and *41) genes into metabolizer phenotypes according to the Clinical Pharmacogenetics Implementation Consortium (CPIC)^18^: PMs with 2 loss-of-function alleles, IMs with 1 wild-type allele and 1 reduced function or null allele, and NMs with 2 fully functioning alleles. *CYP2D6* UMs are described as carrying multiple copies of functional alleles, whereas *CYP2C19* UMs are those with the *17/*17 genotype. For *CYP2C19*, CPIC defines a fifth metabolizer phenotype, RMs, characterized by the presence of a gain of function allele (CYP2C19*17) together with a normal function allele.^18^ The predicted metabolizer phenotype based on *CYP2C19* and *CYP2D6* genotype is reported in Supplemental Table S3.

Six SNPs in the *ABCB1* gene were genotyped, including rs1045642 (C3435T), rs1128503 (C1236T), rs2032582 (G2677T/A), rs2032583, rs2235015, and rs2235040. All *ABCB1* SNPs were checked for Hardy–Weinberg equilibrium deviations in participants of self-reported European ancestry, as they comprised the largest ancestral group. Haploview software version 4.2 (http://www.broad.mit.edu/personal/jcbarret/haploview/) was used to determine the Hardy–Weinberg equilibrium deviation, linkage disequilibrium (LD) (*D*′), and correlation (*r*^2^) among *ABCB1* SNPs.

### Statistical Analyses

Statistical analyses were performed using R Version 4.2.2 (R Foundation for Statistical Computing Platform, 2022). The Shapiro–Wilk test was used to assess the normality of data distribution. The *χ*^2^, Fisher's exact, Mann–Whitney *U*, and Kruskal–Wallis tests were performed for comparisons of demographic and clinical characteristics between ESC-Only and ESC + ARI treatment arms. Given the limited sample size, IM and PMs were pooled together into 1 category, IM + PMs, for *CYP2C19* and *CYP2D6*. RMs and UMs were combined as RM + UMs for *CYP2C19*. Since the sample only included 2 *CYP2D6* UMs, they were excluded from all analyses involving *CYP2D6*.

Phases I and II were analysed separately given the introduction of ARI augmentation for week 8 nonresponders during Phase II. Furthermore, analyses for Phase II were conducted by treatment arms, ESC-Only and ESC + ARI. The effects of *CYP2C19* and *CYP2D6* metabolizer groups and *ABCB1* genotypes on the primary outcomes, change in SS and SF for weeks 0–8 and weeks 8–16, were assessed using repeated measures linear mixed-effects models. Age, sex, ancestry (i.e., European or non-European), and comorbidity with medical condition(s) affecting SF and/or SS (i.e., cardiac, endocrine, gastrointestinal, hepatobiliary, immune system, nervous system, psychiatric, renal and urinary, reproductive system and breast, and rheumatic disorders) were included as fixed effects, and subject and recruitment site were included as random effect factors. Further, to take into consideration the effects of disease severity on sexual dysfunction, we included an interaction between time and total MADRS scores during the corresponding phase as a fixed effect. For ESC + ARI, since there was variability in ARI dosage between participants, it was also included as a fixed effect variable for this treatment arm. We explored whether measures of serum concentrations at week 2 and week 16 were associated with SF and SS change from weeks 0–8 during Phase I and from weeks 8–16 during Phase II using Spearman's rank correlation.

We performed post hoc mediation analyses to assess whether any significant effects of *CYP2C19* or *CYP2D6* metabolizer groups on the SF or SS are mediated by dose-adjusted ESC serum concentrations according to the methods of Baron and Kenney^37^ and MacKinnon et al.^38^ A detailed description of the mediation analyses is provided in Supplemental Figure S1.

Due to the heterogeneity in ancestry among study participants, which may obscure true associations, all analyses were repeated in the largest homogenous ancestral group in the sample, Europeans (*n* = 129, age ranging from 18 to 61, 57.4% female).^39^ To explore the influence of self-reported sex on the relationship between our gene variants and treatment-emergent changes in SS and SF, we conducted all analyses in males and females, separately.

The false discovery rate approach was applied to adjust for multiple comparisons of the influence of the gene variants on the primary outcomes, change in SF and SS, in the analysis of each subsample (i.e., total sample for Phase I and by treatment arms for Phase II), where *q *<* *0.05 (two-tailed) was considered significant.^40,41^ For exploratory comparisons (e.g., assessment of baseline sample characteristics), *p *< 0.05 was considered significant

## Results

### Sample Characteristics

Table 1 summarizes the demographic and clinical characteristics of the sample. Prior to week 8, 31 participants dropped out and were not included in the analyses due to missing SexFX scores and drug serum level data. Based on drug serum levels, all participants were adherent to treatment at Phase I (*n* = 178), while 7 participants were considered nonadherent during Phase II due to a lack of treatment medication detected in blood at both week 10 and week 16; therefore, these participants were excluded from Phase II analyses, leaving *n* = 171 during this phase. Nonadherence was not associated with *CYP2C19* or *CYP2D6* metabolizer groups nor with *ABCB1* genotypic groups.

**Table 1.** CAN-BIND-1 Sample Demographics and Clinical Information.

Characteristics	Phase I (*N* = 178)	Phase II: ESC-Only (*N* = 80)	Phase II: ESC + ARI (*N* = 91)	
	*N* (%)	*N* (%)	*N* (%)	*p* value^a^
Sex				
Females	110 (61.8%)	53 (66.3%)	54 (59.3%)	0.439
Ancestry^b^				0.614
European	129 (72.5%)	57 (71.2%)	69 (75.8%)	
Non-European [African (4), East Asian (14), Latin American (9), South Asian (5), South East Asian (4), and mixed ancestry (13)]	49 (27.5%)	23 (28.8%)	22 (24.2%)	
*CYP2C19*				0.326
NM	71 (39.9%)	32 (40.0%)	37 (40.7%)	
IM	51 (28.7%)	22 (27.5%)	27 (29.7%)	
PM	5 (2.8%)	3 (3.8%)	1 (1.1%)	
RM	43 (24.2%)	21 (26.3%)	20 (22.0%)	
UM	7 (3.9%)	1 (1.3%)	6 (6.5%)	
*CYP2D6*				0.317
NM	99 (55.6%)	40 (50.0%)	54 (59.3%)	
IM	60 (33.7%)	30 (37.5%)	28 (30.8%)	
PM	11 (64.7%)	5 (6.3%)	6 (6.6%)	
*ABCB1* rs1045642 (C3435T)				0.694
TT	52 (29.2%)	21 (26.3%)	29 (31.9%)	
CT	78 (43.8%)	37 (46.2%)	38 (41.8%)	
CC	47 (26.4%)	22 (27.5%)	23 (25.3%)	
*ABCB1* rs1128503 (C1236T)				0.987
CC	48 (27.0%)	23 (28.4%)	23 (23.7%)	
CT	83 (46.6%)	39 (48.1%)	41 (42.3%)	
TT	31 (17.4%)	14 (17.3%)	15 (15.5%)	
*ABCB1* rs2032582 (G2677T/A)				0.661
GG	45 (25.3%)	23 (28.8%)	21 (23.1%)	
GT/GA	87 (48.9%)	39 (48.8%)	45 (49.5%)	
TT/TA/AA	43 (25.2%)	17 (21.3%)	23 (25.3%)	
*ABCB1* rs2032583				0.477
TT	142 (79.8%)	66 (82.5%)	70 (76.9%)	
CT/CC	36 (20.2%)	14 (17.5%)	21 (23.1%)	
*ABCB1* rs2235015				0.452
GT/TT	58 (32.6%)	29 (36.2%)	27 (29.7%)	
GG	120 (67.4%)	51 (63.8%)	64 (70.3%)	
*ABCB1* rs2235040				0.371
GG	130 (73.0%)	64 (79.0%)	60 (61.9%)	
AA/AG	33 (18.5%)	13 (16.0%)	19 (19.6%)	

	Mean (SD)	Mean (SD)	Mean (SD)	*p* value^b^
Age (years)	35.4 (12.8)	34.8 (12.4)	36.2 (13.1)	0.511
Baseline MADRS score	30.0 (5.5)	29.1 (5.4)	31.0 (5.5)	**0**.015*
SexFX scores	At Baseline	At Week 16	At Week 16	
Sexual function	19.2 (10.8)	21.4 (11.6)	19.4 (9.8)	0.160
Desire	6.2 (4.8)	7.2 (5.0)	5.3 (4.0)	**0**.030*
Arousal	8.0 (3.5)	8.7 (3.8)	8.3 (3.2)	0.455
Orgasm	5.2 (4.0)	5.6 (4.4)	5.9 (4.0)	0.881
Preorgasm	14.3 (7.8)	16.0 (8.0)	13.7 (6.4)	0.085
Sexual satisfaction	8.5 (5.8)	11.2 (6.0)	8.1 (5.9)	**0**.001*

*Note*. ARI = aripiprazole; ESC = escitalopram; IM = intermediate metabolizer; NM = normal metabolizer; PM = poor metabolizer; RM = rapid metabolizer; SF = sexual function; SS = sexual satisfaction; SD = standard deviation; UM = ultrarapid metabolizer.

^a^ Fisher's exact test or chi-square test for categorical variables and Wilcoxon rank sum test for continuous variables, as appropriate, for comparisons between the two treatment arms, ESC-Only and ESC + ARI.

^b^ Categories for ancestry are adapted from the International Genome Sample Resource 1000 Genomes Project (http://www.internationalgenome.org/category/population/)

*p < 0.05.

At the start of Phase I, total MADRS score was negatively correlated with SF (*r* = −0.25, *p *< 0.001) and SS (*r* = −0.24, *p *= 0.002) at baseline, with participants with higher MADRS score (i.e., greater severity of depression) reporting more sexual dysfunction. When comparing Phase II treatment arms, ESC-Only and ESC + ARI differed significantly in the SF domain, desire (*W* = 3826.5, *p *= 0.023), and SS (*W* = 4070.5 *p *= 0.001), with ESC + ARI reporting experiencing lower desire (ESC + ARI: mean = 5.4 ± 3.9 vs. ESC-Only: mean = 7.3 ± 5.0) and SS (ESC + ARI: mean = 8.1 ± 5.9 vs. ESC-Only: mean = 11.3 ± 6.0) compared to ESC-Only. There were no statistically significant baseline differences between treatment arms with respect to other demographic characteristics, SexFX scores, and *CYP2C19* and *CYP2D6* metabolizers and *ABCB1* genotypic groups.

For all 6 *ABCB1* SNPs tested, genotype distributions were in Hardy–Weinberg equilibrium in the European subsample (*N* = 129) (Supplemental Table S4). Some variants, rs2032583, rs2032582, rs2235040, and rs1128503, were in strong LD (*D*′ = 0.94–1, *r*^2^ = 0.84–1).

### Associations of CYP2C19, CYP2D6, and ABCB1 with Treatment-Related Changes in SF and SS

**Figure 2.**
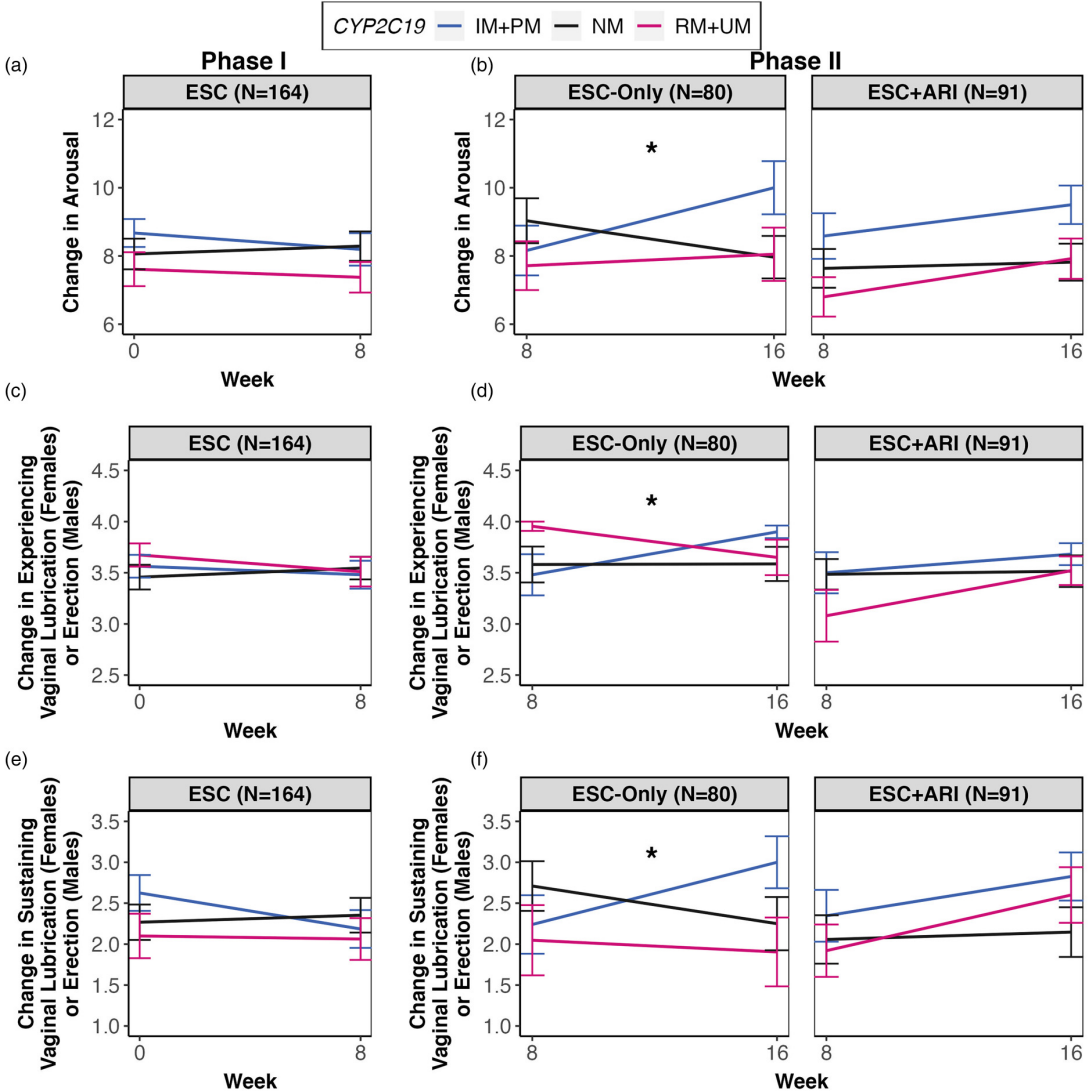
Treatment-related change in sexual arousal over time for Phases I and II by *CYP2C19* metabolizer groups. (a) During Phase I, no associations were observed between *CYP2C19* metabolizer groups and treatment-related changes in sexual arousal from weeks 0–8. (b) During Phase II, in the ESC-Only treatment arm, *CYP2C19* IM + PMs showed improvements in sexual arousal relative to NMs, whereas NMs showed a decline in sexual arousal from weeks 8–16 (*B* = 0.44, 95% CI, 0.21 to 0.67). This effect was not observed in the ESC + ARI treatment arms. (c) No associations between *CYP2C19* and changes in having vaginal lubrication in females and erection in males with treatment were observed during Phase I. (d) *CYP2C19* IM + PMs reported less difficulty with having vaginal lubrication in females and erection in males between weeks 8 and 16 compared to NMs (*B* = 0.09, 95% CI, 0.03 to 0.16), which was observed only in ESC-Only. (e) There were no associations between *CYP2C19* and changes in sustaining vaginal lubrication in females and erection in males with treatment during Phase I. (f) *CYP2C19* IM + PMs in the ESC-Only treatment arm reported less difficulty with sustaining vaginal lubrication in females and erection in males between weeks 8 and 16 compared to NMs (*B* = .17, 95% CI, −0.03 to 0.18). All linear mixed-effects analyses included age, ancestry, sex, comorbidity with medication conditions that affect SF/SS, total MADRS scores during the corresponding phase, *CYP2C19* and *CYP2D6* metabolizer groups as fixed effects, and individual and recruitment site as random effects. For ESC + ARI, ARI dosage was included as a fixed effect.

*Note*. ARI = aripiprazole; CI = confidence interval; ESC = escitalopram; IM = intermediate metabolizer; NM = normal metabolizer; PM = poor metabolizer; RM = rapid metabolizer; UM = ultrarapid metabolizer. *Indicates between-group differences with *q* < 0.05.

During Phase I, there were no significant differences in SF or SS change between *CYP2C19* and *CYP2D6* metabolizer and *ABCB1* genotypic groups. During Phase II, there was a significant association between the *CYP2C19* metabolizer group and treatment-related change in sexual arousal in the ESC-Only treatment arm following multiple testing corrections, *F*(2,54) = 8.00, *p *< 0.001, *q *= 0.048, whereas ESC + ARI did not demonstrate this association (Supplemental Table S5). Repeated measures linear mixed-effects analysis revealed that *CYP2C19* IM + PMs (*n* = 25) showed improvements in sexual arousal from weeks 8–16 (*B* = 0.44, 95% confidence interval [CI], 0.21 to 0.67), while RM + UMs (*n* = 22) did not differ significantly from NMs (*n* = 32) (Figure 2b). More specifically, *CYP2C19* IM + PMs reported less difficulty with having (*B* = 0.09, 95% CI, 0.03 to 0.16) and sustaining (*B* = 0.17, 95% CI, −0.03 to 0.18) vaginal lubrication in females and erection in males between weeks 8 and 16 compared to NMs (Figure 2d and f). This association was also observed in the European subsample, which showed ESC-Only *CYP2C19* IM + PMs improving in functions related to sexual arousal compared to NMs with treatment, *F*(2,40) = 11.18, *p *< 0.001, *q *< 0.001.

Since sexual arousal is experienced differently between females and males, we conducted post hoc analyses stratifying by sex. We observed a significant association between the *CYP2C19* metabolizer group and sexual arousal in both females, *F*(2,32) = 3.65, *p *= 0.037, and males, *F*(2,16) = 7.14, *p *= 0.006, in the same direction. Follow-up mediation analyses revealed that there was a lack of evidence for a significant mediating effect by dose-adjusted ESC or S-desmethylcitalopram (S-DCT) serum concentrations on the relationship between the *CYP2C19* metabolizer group and change in sexual arousal with treatment.

The following analyses on the effects of an interaction between all three genes, *CYP2C19*, *CYP2D6*, or *ABCB1*, on treatment-related changes in SF and SS over time were exploratory.^42^ Interestingly, there was a significant effect of an interaction between *CYP2C19* and *CYP2D6* metabolizer groups and *ABCB1* SNP, rs1045642, on SF change, *F*(4,131) = 4.52, *p *= 0.002, *q* = 0.048. Simple effects analyses revealed that significantly greater mean decline in SF from baseline was observed in *CYP2D6* and *CYP2C19* IM + PMs who had the CC (*B* = −4.37, 95% CI, −7.09 to −1.63) or CT (*B* = −2.65, 95% CI, −4.94 to −0.36) genotype at the *ABCB1* rs1045642 SNP or was *CYP2D6* IM + PM and *CYP2C19* RM + UM and had the rs1045642 CC genotype (*B* = −5.54, 95% CI, −8.25 to −2.82) compared to those with normal CYP function and P-gp expression (i.e., NMs of *CYP2D6* and *CYP2C19* with the rs1045642 TT genotype) (Supplemental Figure S2a). Likewise, the same effect was observed for the arousal and preorgasm subdomains of SF (Supplemental Figure S2b and c).

### Associations of ESC and ARI Exposure with SF and SS

Supplemental Table S6 presents the Spearman correlations between the outcome measures, SF and SS, and serum concentrations of ESC and its metabolite, S-desmethylcitalopram (S-DCT), and ARI and its metabolite, dehydroaripiprazole. No significant associations were observed between these variables following multiple testing corrections.

**Figure 3.**
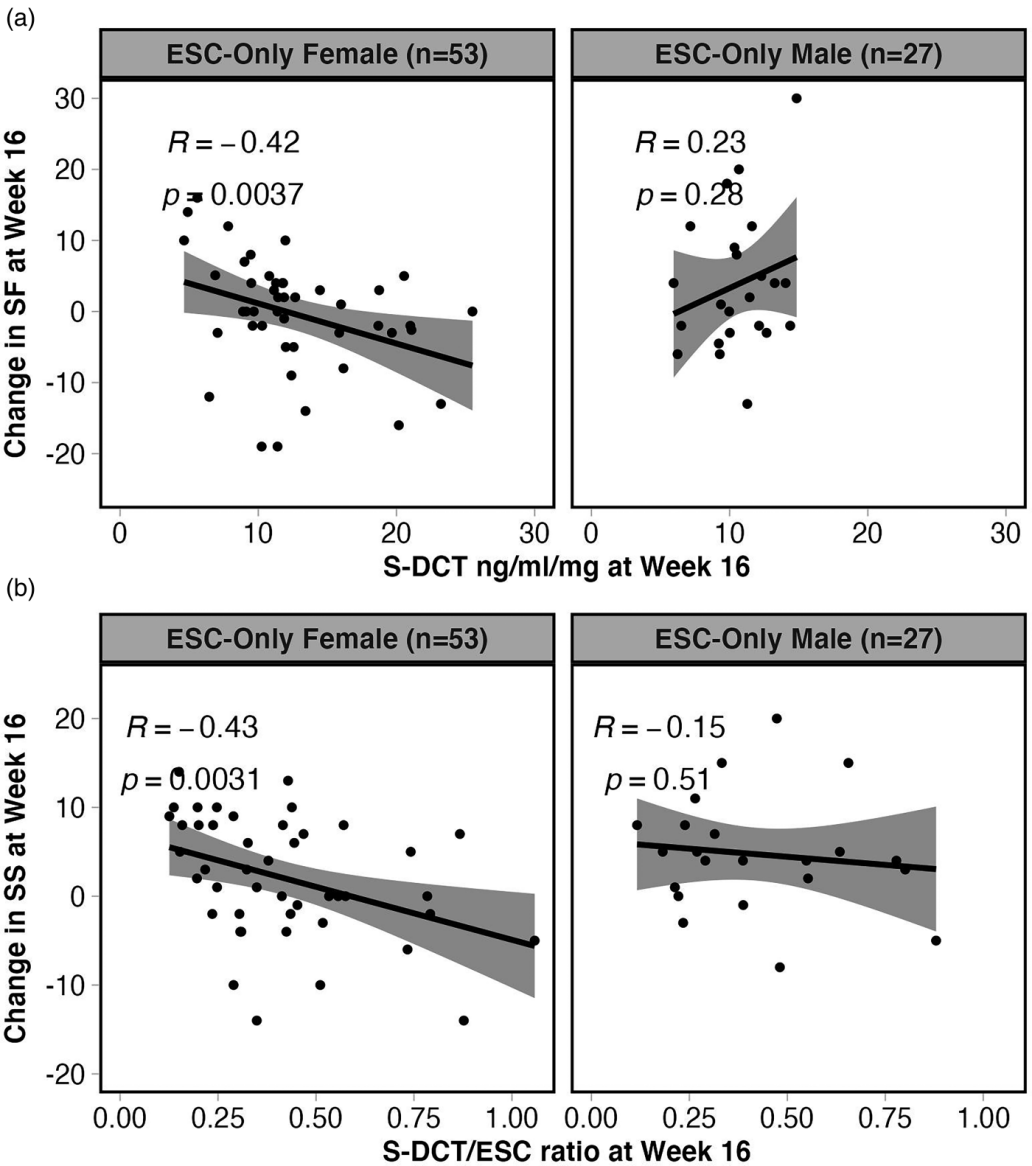
Spearman correlations between concentrations of ESC metabolite, S-DCT, and S-DCT/ratio in serum and SF and SS at Week 16, respectively, in females and males of the ESC-Only treatment arm. (a) ESC-Only females with higher S-DCT demonstrated more decline in SF (*r* = −0.42, *p *= 0.004, *q *= 0.034), whereas males did not show a significant correlation between these variables. Furthermore, (b) only females in this treatment arm with a higher S-DCT/ESC ratio (i.e., higher S-DCT relative to ESC) demonstrated more decline in SS (*r* = −0.43, *p *= 0.003, *q *= 0.034).

*Note*. ESC = escitalopram; S-DCT = S-desmethylcitalopram; SF = sexual function; SS = sexual satisfaction.

When analysing males and females separately, interestingly, ESC-Only females showed a significant correlation between concentrations of S-DCT and S-DCT/ESC ratio in serum and SF and SS at week 16, respectively. ESC-Only females with higher S-DCT and S-DCT/ESC ratio (i.e., higher S-DCT relative to ESC) demonstrated more decline in SF (*r* = −0.42, *p *= 0.004, *q *= 0.034) and SS (*r* = −0.43, *p *= 0.003, *q *= 0.034), respectively (Figure 3). ESC-Only females also demonstrated a trend for a correlation between S-DCT and change in sexual arousal in the same direction (*r* = −0.39, *p *= 0.009, *q *= 0.052). These correlations were not observed in males.

## Discussion

To our knowledge, this is the first study to investigate the effects of *CYP2C19* and *CYP2D6* metabolizer phenotypes on changes in SF and SS related to the treatment of MDD with ESC.^43^ Although the exact mechanism of SSRI-induced sexual dysfunction is not well understood, it is postulated that elevated levels of synaptic 5-HT and increased activity of 5-HT_2A_ and 5-HT_2C_ receptors induced by SSRIs exacerbate the inhibition of circuitry underlying sexual behaviours and reflexes.^35,44^ Therefore, we hypothesized that those with gene variants contributing to elevated brain SSRI concentrations (i.e., *CYP2C19* and *CYP2D6* IM + PMs and *ABCB1* genotypes associated with lower P-gp function or expression) are expected to have increased 5-HT neurotransmission enhancing the inhibition of brain circuitry mediating SF and SS, and thus will experience a decline in SF and SS from baseline with treatment.

Interestingly, we did not observe any significant associations between *CYP2D6* and *ABCB1* with SF or SS, and the association we observed between *CYP2C19* and change in sexual arousal was not in the direction we had predicted. During Phase II, in the ESC-Only treatment arm, it was *CYP2C19* IM + PMs who demonstrated the most treatment-related *improvements* in sexual arousal from weeks 8–16, whilst NMs showed relatively more *decline* in sexual arousal. Specifically, *CYP2C19* IM + PMs reported a higher frequency of having and sustaining vaginal lubrication in females and erection in males.

The mechanisms by which *CYP2C19* NMs of the ESC-Only treatment arm experienced more decline in sexual arousal relative to IM + PMs may in part be explained by ESC serum levels and its metabolite. Although measuring brain levels of ESC and S-DCT is not feasible, serum concentrations can be used as a proxy to investigate the effects of these compounds on SF and SS. Our serum level analyses revealed a decline in SF, particularly sexual arousal, with higher S-DCT concentrations in serum of females of the ESC-Only treatment arm, which was not observed in males. A previous study reported a decrease in *CYP2C19* activity with oral contraceptive (OC) usage in females compared to females who did not use OCs.^45^ Therefore, OC usage may be confounding these findings, as OC-using females may have slower ESC metabolism, and therefore lower S-DCT concentrations, than non-OC-using females. Since our study did not collect data on OC usage, further research with more comprehensive data on OC usage and timing of menstrual cycles of female participants on antidepressants is warranted.

S-DCT is itself active as a 5-HT reuptake inhibitor with potency comparable to the parent compound. Thus, it is possible that S-DCT contributes to the physiological blockade of 5-HT transport leading to elevated synaptic 5-HT and enhanced inhibition of neural pathways relating to sexual functioning.^46,47^ Furthermore, *ex vivo* assays using serum and brain tissue extracts collected from mice injected intraperitoneally with citalopram (CIT) or desmethylcitalopram (DCT) at different time points have shown that DCT levels increased in the brain whereas CIT concentrations decreased.^46^ The accumulation of DCT in the brain over time suggests that DCT may have a longer-lasting effect on SF than CIT.

**Figure 4.**
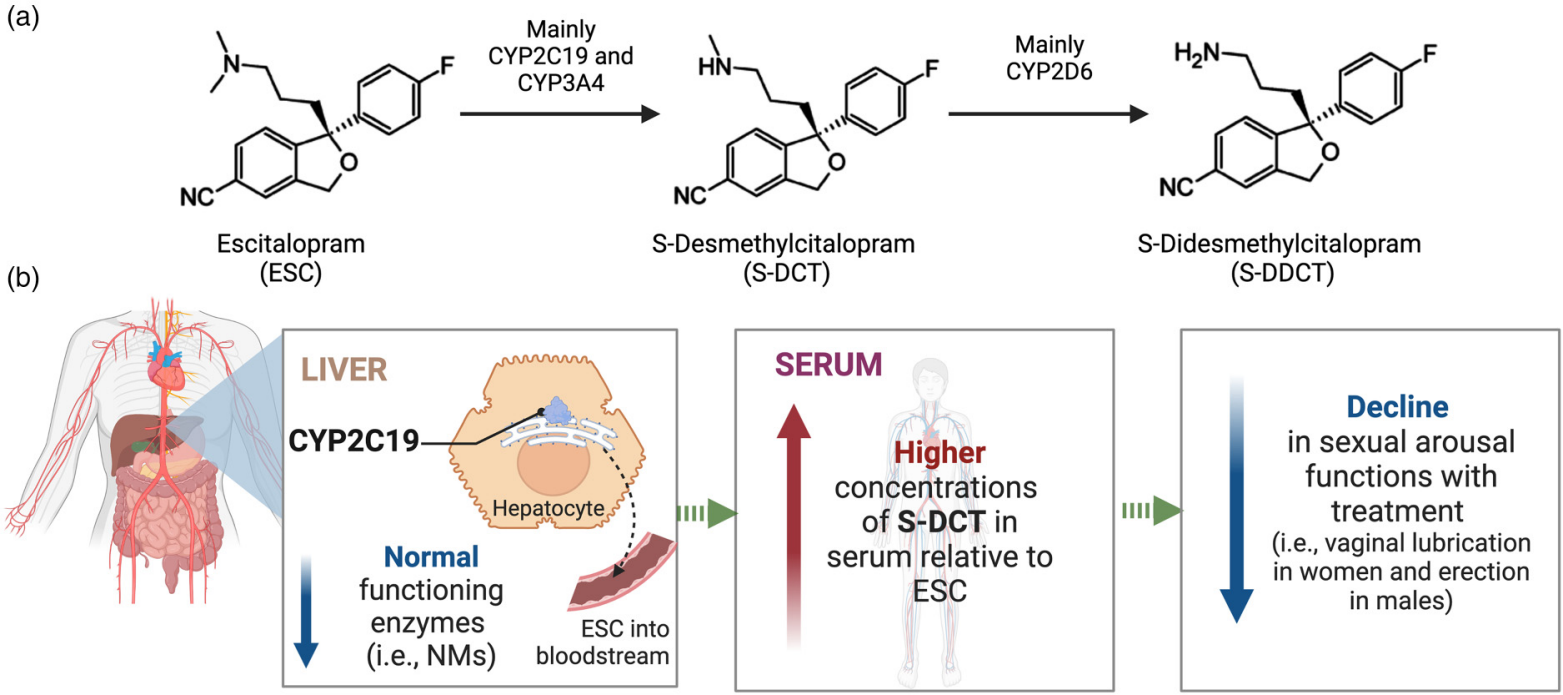
Proposed model explaining the effects of variations in *CYP2C19* on sexual arousal functions. (a) ESC biotransformation pathway. (b) Genetic differences in the metabolism of ESC by *CYP2C19* are expected to contribute to variations in treatment-emergent dysfunctions in sexual arousal that some patients on SSRIs experience. Specifically, individuals with gene variants contributing to elevated brain concentrations of S-DCT (i.e., *CYP2C19* NMs), which itself is active as a serotonin reuptake inhibitor with potency comparable to the parent compound, are expected to have increased serotonin neurotransmission leading to enhanced inhibition of brain circuitry mediating sexual arousal. As a result, these individuals may experience more negative effects of the medication on their ability to have and sustain vaginal lubrication in females and erection in males.

*Note*. ESC = escitalopram; NM = normal metabolizer; S-DCT = S-desmethylcitalopram.

We have previously shown that in those on ESC monotherapy, *CYP2C19* NMs demonstrated approximately twice as high S-DCT/ESC ratios compared to IM + PMs, which was observed even when stratified by sex.^21^ This indicates that NMs have higher serum concentrations of S-DCT relative to ESC, whereas *CYP2C19* IM + PMs, who have a reduced ability to metabolize ESC, demonstrate lower metabolite levels relative to the parent compound (Supplemental Figure S4). These findings taken together suggest that a possible explanation for deteriorations in sexual arousal observed in *CYP2C19* NMs may be due to elevated concentrations of S-DCT relative to ESC in these individuals given S-DCT's affinity for the 5-HT transporter, as well as accumulation in the brain over time, whereas IM + PMs, lacking high concentrations of S-DCT, do not show this deterioration (see Figure 4 for the proposed model). It is important to note that when we assessed the mediating effects of serum levels of ESC, S-DCT, and the S-DCT/ESC ratio on the relationship between *CYP2C19* metabolizer groups and change in sexual arousal, drug serum measures did not appear to be mediating this effect. Therefore, further research is required to validate these findings in a larger, independent sample and elucidate the effects of S-DCT on the mechanisms underlying treatment-emergent sexual dysfunction.

Since sexual impairments are also a commonly experienced symptom of MDD, self-reported sexual dysfunction during the trial is unlikely to be solely due to the pharmacological treatment or genetic variations in the studied genes. The advantages of the present study include the availability of longitudinal data on SF, SS, and MADRS, which allowed us to assess the trajectory of treatment-related changes in these variables, as well as control for variability between participants. Some limitations of this study that are worth noting include (a) a limited sample size, (b) numerous factors influencing the reporting and experience of SF and SS, (c) lack of data on concurrent medications and week 8 serum levels, and (d) the limited generalizability of the findings to non-European populations. First, the sample size is limited, especially during Phase II when analysis is conducted by treatment arm, which limits our power to detect clinically significant differences in SF and SS between metabolizer or genotypic groups. Second, reporting of SF and SS may be affected by numerous individual factors, such as age, sex, levels of gonadal steroid hormones and timing of menstrual cycle,^48,49^ OC usage in females, stability of relationships, level of sexual activity, sexual preferences, and cultural differences or individual openness to answering questions on one's sexual life, which we did not control for. Third, comedications were not recorded which might have modulated *CYP2C19* or *CYP2D6* activity and resulted in phenoconversion. Comedications, such as hormonal contraceptives, may also potentially influence SF/SS.^50^ Furthermore, for the analyses of associations of ESC and ARI exposure with SF and SS, week 2 serum levels were used as a proxy, since blood was not collected on week 8 when SexFX was administered, whereas for week 16, both serum levels and SexFX scores were available. Finally, our sample was ∼72% European, which limits the generalizability of the findings to other ancestral groups, given that *CYP2C19*, *CYP2D6*, and *ABCB1* allele frequencies differ between populations. Particularly, the number of CYP2D6 UMs was lower than expected within this sample (*n* = 2), and as a result, this group could not be included in our analyses. *CYP2D6* UM phenotype is present in ∼28% of North Africans, Ethiopians, and Arabs, 3% of Europeans, 3% of African Americans, and 1% of Hispanics, Chinese, and Japanese.^51,52^ Therefore, further research examining antidepressant efficacy and changes in SF in *CYP2D6* UMs is necessary.

## Conclusions

To our knowledge, this is the first study to examine treatment-emergent sexual dysfunction in individuals on ESC with ARI augmentation. We showed that genetic variations within *CYP2C19* appear to be related to changes in sexual arousal induced by treatment with ESC monotherapy. The results showed that *CYP2C19* NMs could be at an increased risk of experiencing SSRI-associated decline in sexual arousal; however, these findings need to be replicated in a larger, independent sample. This study sheds some light on the pharmacogenetics of SSRI-related sexual dysfunction, which has been understudied, even though it plays a major role in treatment adherence, and ultimately, the efficacy of SSRIs for individuals with MDD.

## Supplemental Material

sj-docx-1-cpa-10.1177_07067437231203433 - Supplemental material for Influence of *CYP2C19*, *CYP2D6*, and *ABCB1* Gene Variants and Serum Levels of Escitalopram and Aripiprazole on Treatment-Emergent Sexual Dysfunction: A Canadian Biomarker Integration Network in Depression 1 (CAN-BIND 1) Study Supplemental material, sj-docx-1-cpa-10.1177_07067437231203433 for Influence of *CYP2C19*, *CYP2D6*, and *ABCB1* Gene Variants and Serum Levels of Escitalopram and Aripiprazole on Treatment-Emergent Sexual Dysfunction: A Canadian Biomarker Integration Network in Depression 1 (CAN-BIND 1) Study by Farhana Islam, Leen Magarbeh, Samar S. M. Elsheikh, Stefan Kloiber, Caroline W. Espinola and Venkat Bhat, Benicio N. Frey, Roumen Milev, Claudio N. Soares, Sagar V. Parikh, Franca Placenza, Stefanie Hassel, Valerie H. Taylor, Francesco Leri, Pierre Blier, Rudolf Uher, Faranak Farzan, Raymond W. Lam, Gustavo Turecki, Jane A. Foster, Susan Rotzinger, Sidney H. Kennedy, Daniel J. Müller in The Canadian Journal of Psychiatry
