# Supplementary material for: Influence of CYP2C19, CYP2D6, and ABCB1 Gene Variants and Serum Levels of Escitalopram and Aripiprazole on Treatment-Emergent Sexual Dysfunction: A Canadian Biomarker Integration Network in Depression 1 (CAN-BIND 1) Study
Source: Can J Psychiatry. 2023 Oct 5;69(3):183–95. doi: 10.1177/07067437231203433 (PMC10874600; doi:10.1177/07067437231203433)
Supplement: sj-docx-1-cpa-10.1177_07067437231203433 - Supplemental material for Influence of CYP2C19, CYP2D6, and ABCB1 Gene Variants and Serum Levels of Escitalopram and Aripiprazole on Treatment-Emergent Sexual Dysfunction: A Canadian Biomarker Integration Network in Depression 1 (CAN-BIND 1) Study [file sj-docx-1-cpa-10.1177_07067437231203433.docx]

**SUPPLEMENTARY MATERIALS**

**Supplementary Methods**

***Serum Concentration Measurements***

During the collection of the blood samples, participants reported the time at which they took their last dosage of the study medication. The quantification of serum ESC, ARI and their respective major metabolites, *S*-desmethylcitalopram (S-DCT) and dehydroaripiprazole (DHA), was developed and performed at our CAMH Clinical Laboratory using LC-MS/MS technology (Centre for Addiction and Mental Health, Toronto, Canada). Briefly, a mix of deuterated internal standards (Cerilliant) was added to 100 µL of each serum specimen, calibrator (Cerilliant) and quality control (MassCheck Antidepressants/Neuroleptics, Level 1 and Level 2). Proteins were precipitated using 300 µL of 9:1 Acetonitrile:Methanol (Sigma-Aldrich) followed by 5 min centrifugation at 9000 rpm. 25 µL of each supernatant was diluted with 200 µL of 0.1% Formic acid (Sigma-Aldrich) and then analyzed on the LC-MS/MS platform consisting of ThermoFisher TSQ Quantum Ultra mass spectrometer coupled with ThermoFisher Surveyor LC pump and HTC PAL autosampler fitted with a Kinetex F5 2.6 µm, 100 x 2.1 mm column (Phenomenex). Six-minute gradient elution with 0.1% Formic Acid (mobile phase A) and Acetonitrile (mobile phase B) was applied. Collision energies ranged from 14 to 20. Mass transitions (M+H) were monitored in SIM mode as follows: ARI (448®285); DHA (446®285); Citalopram (325 ®109); Desmethyl-citalopram (314 ®109) Quantification was performed against a 7-point calibration curve ranging from 10 to 1000 ng/mL for each analyte. The assay limit of detection was 5 ng/mL. Vendor nominal means for quality controls were used to assess the assay accuracy for each of the four analytes at Level 1 and Level 2, respectively, and ± 15 % of nominal mean value combined with <15 % analytical precision was considered acceptable assay performance. Both the laboratory means and the assay precisions were within the set acceptance criteria. Data was analyzed with ThemoFisher XCalibur software.

**Table S1.** Items on the Sex FX questionnaire.

| **Item*** | **Question text** | **Gender specific wording** | **Sexual Domain** |
| --- | --- | --- | --- |
| 1. | Experience a desire for sexual activity | No | Desire |
| 2. | Fantasize about sexual activity | No | Desire |
| 3. | Have an interest in initiating sexual activity | No | Desire |
| 4. | Have the ability to feel sexually excited ("turned on") | No | Desire |
| 5. | Have difficulty with vaginal lubrication [female]  Have difficulty getting an erection [male] | Yes | Arousal |
| 6. | Become aroused without being touched [female]  Have an erection without being touched [male] | Yes | Arousal |
| 7. | Engage in sexual activity (intercourse or masturbation) | No | Arousal |
| 8. | Have difficulty staying lubricated during sexual activity [female]  Have difficulty staying erect during sexual activity [male] | Yes | Arousal |
| 9. | Experience an orgasm | No | Orgasm |
| Or | Take longer than you would like to have an orgasm [female]  Take longer than you would like to ejaculate [male] | Yes | Orgasm |
| 11. | Fail to have an orgasm during sexual activity [female]  Fail to ejaculate during sexual activity [male] | Yes | Orgasm |
| 12. | Satisfaction with your sexual functioning | No | Satisfaction |
| 13. | Enjoyment of your sexual romantic life | No | Satisfaction |

*Items 1-11 are on a 5-point Likert scale, whereas items 12-13 are on a 10-point scale.

**Table S2**. TaqMan® genotyping assay IDs for *CYP2C19* and *CYP2D6*.

| **Gene** | **rs#** | **Assay ID** |
| --- | --- | --- |
| *CYP2C19* | rs12248560 | C____469857_10 |
| *CYP2C19* | rs4244285 | C__25986767_70 |
| *CYP2C19* | rs4986893 | C__27861809_10 |
| *CYP2D6* | CNVInt6 | Hs04502391_cn |
| *CYP2D6* | CNVx9 | Hs00010001_cn |
| *CYP2D6* | rs1065852 | C__11484460_40 |
| *CYP2D6* | rs16947 | C__27102425_10 |
| *CYP2D6* | rs28371706 | C___2222771_40 |
| *CYP2D6* | rs28371725 | C__34816116_20 |
| *CYP2D6* | rs35742686 | C__32407232_50 |
| *CYP2D6* | rs3892097 | C__27102431_D0 |
| *CYP2D6* | rs5030655 | C__32407243_20 |
| *CYP2D6* | rs5030656 | C__32407229_60 |
| *CYP2D6* | rs59421388 | C__34816113_20 |

**Table S3.** Distribution of *CYP2C19* and *CYP2D6* genotypes and predicted metabolizer status.

|  | **Frequency (*n,* %)** | | |  |  |
| --- | --- | --- | --- | --- | --- |
| **Metabolizer Status** | **Sample**  **N=178** | **ESC**  **N=81** | **ESC+ARI**  **N=97** | **Age**  **(mean ± SD)** | **Genotypes (*n*)** |
| ***CYP2C19*** | | | | | |
| NM | 71 (39.89) | 33 (40.74) | 38 (39.18) | 35.04 ± 12.96 | *1/*1 (71) |
| IM | 51 (28.65) | 22 (27.16) | 29 (29.90) | 35.67 ± 12.74 | *1/*2 (39)  *2/*17 (10) |
|  |  |  |  |  | *1/*3 (1)  *3/*17 (1) |
| PM | 5 (2.809) | 3 (3.704) | 2 (2.062) | 34.20 ± 14.20 | *2/*2 (3) |
|  |  |  |  |  | *2/*3 (2) |
| RM | 43 (24.16) | 21(25.93) | 22 (22.68) | 35.91 ± 13.08 | *1/*17 (43) |
| UM | 7 (3.933) | 1 (1.235) | 6 (6.186) | 36.14 ± 12.54 | *17/*17 (7) |
| Not known | 1 (0.57) | 1 (1.235) | 0 (0) | - | - |
| ***CYP2D6*** | | | | | |
| NM | 99 (55.62) | 41 (50.61) | 58 (59.79) | 36.61 ± 13.18 | *1/*2 (27) |
|  |  |  |  |  | *1/*1 (22) |
|  |  |  |  |  | *2/*4 (10) |
|  |  |  |  |  | *2/*41 (9) |
|  |  |  |  |  | *2/*2 (7) |
|  |  |  |  |  | *1/*41 (5) |
|  |  |  |  |  | *1/*36+*10 (4) |
|  |  |  |  |  | *2/*5 (4) |
|  |  |  |  |  | *1/*9 (3) |
|  |  |  |  |  | *1/*17 (2) |
|  |  |  |  |  | *1/*10 (1) |
|  |  |  |  |  | *2/*3 (1) |
|  |  |  |  |  | *2/*36+*4 (1) |
|  |  |  |  |  | *2/*9 (1) |
|  |  |  |  |  | *2/*9 (xN) (1) |
|  |  |  |  |  | *2/*17 (1) |
| IM | 60 (33.71) | 30 (37.04) | 30 (30.93) | 33.70 ± 12.86 | *1/*4 (23) |
|  |  |  |  |  | *1/*5 (8) |
|  |  |  |  |  | *36+10/*36 (5) |
|  |  |  |  |  | *1/*4 (xN) (3)  *10/*36+10 (3) |
|  |  |  |  |  | *41/*41 (3) |
|  |  |  |  |  | *1/*3 (2) |
|  |  |  |  |  | *4/*41 (2) |
|  |  |  |  |  | *4/*10 (2) |
|  |  |  |  |  | *2/*4 (xN) (1) |
|  |  |  |  |  | *4/*9 (1) |
|  |  |  |  |  | *4/*36+*10 (1) |
|  |  |  |  |  | *5/*9 (1) |
|  |  |  |  |  | *5/*36+*10 (1) |
|  |  |  |  |  | *6/*41 (1) |
|  |  |  |  |  | *9/*9 (1) |
|  |  |  |  |  | *10/*41 (1) |
|  |  |  |  |  | *36+*10/*41 (1) |
| PM | 11 (6.18) | 5 (6.173) | 6 (6.186) | 38.45 ± 10.14 | *4/*4 (7) |
|  |  |  |  |  | *4/*5 (2) |
|  |  |  |  |  | *4/*6 (1) |
|  |  |  |  |  | *5/*6 (1) |
| UM* | 2 (1.124) | 2 (2.469) | 0 (0) | 35.50 ± 14.85 | *1/*2(xN) (1) |
|  |  |  |  |  | *2/*2(xN) (1) |
| Not known | 6 (3.371) | 3 (3.704) | 3 (3.093) | 27.67 ± 3.78 | - |

**Note*: UMs for CYP2D6 were excluded from analyses due to the small sample size (n=2).

IM = Intermediate Metabolizer; NM = Normal Metabolizer; PM = Poor Metabolizer; RM = Rapid Metabolizer; SD = Standard error; UM = Ultrarapid Metabolizer.

**Figure S1.** Diagram depicting the effects being tested through mediation analyses.


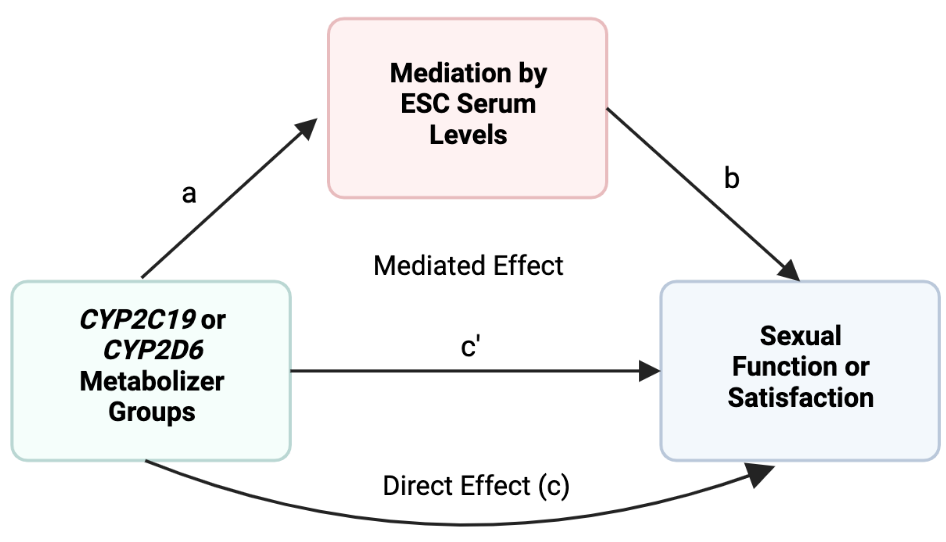


The variables included in the mediation analyses were sexual function (SF), or its subdomains, desire, arousal, orgasm, and pre-orgasm, or satisfaction (SS) as the dependent (y) variable, *CYP2C19* or *CYP2D6* metabolizer groups as the independent (x) variable, and ESC serum levels as the mediator (m) variable. The direct effect (c) refers to the effect of *CYP2C19* or *CYP2D6* metabolizer groups on SF or SS. This is hypothesized to be mediated through the effects of *CYP2C19* or *CYP2D6* metabolizer groups on ESC serum levels (a) and subsequent effects of ESC serum levels on SF and SS (b). Through this mediation effect, the relationship between *CYP2C19* or *CYP2D6* metabolizer groups and SF or SS would be considered an indirect effect (c’). Sequential linear mixed effects models including fixed effects, age, sex, ancestry, comorbidity with a medical condition affecting SF or SS, and disease severity (i.e., MADRS during the corresponding phase), and random effects, individual and recruitment site, were conducted to obtain the values of a, b, c, and c’. A complete mediation is present if c but not c’ is statistically significant, and a partial mediation is present if both c and c’ are statistically significant. R package “Mediation” was used to perform nonparametric bootstrap analyses with 5000 iterations to calculate the estimate of the mediated effect, which was considered significant when the 95% confidence interval (CI) excluded zero at *p*<0.05.

**Table S4.** Position, Allelic Distribution and Role of *ABCB1* Single-Nucleotide Polymorphisms

| **dbSNP ID** | **Position^a^** | **Minor allele**  **(Major allele)** | **MAF** | **Hardy-Weinberg p^b^** | **Role** |
| --- | --- | --- | --- | --- | --- |
| rs1045642 | 87509329 | C(T) | 0.49 | 1.000 | Exon 27 |
| rs1128503 | 87550285 | T(C) | 0.45 | 0.596 | Exon 13 |
| rs2032582 | 87531302 | T/A(G) | 0.44/ 0.05 | 0.381 | Exon 22 |
| rs2032583 | 87531245 | C(T) | 0.11 | 0.482 | Intron 22 |
| rs2235015 | 87570248 | T(G) | 0.19 | 0.815 | Intron 5 |
| rs2235040 | 87536434 | A(G) | 0.11 | 0.717 | Intron 21 |

^a^ Relative position on chromosome 7 are taken from the National Center for Biotechnology Information, genome build 38.

^b^ No deviation from Hardy-Weinberg equilibrium in the European subsample.

MAF: Minor allele frequency.

**Table S5**. Summary of mixed effects models for change in sexual arousal functioning during Phase I and II adjusted for age, ancestry, sex, MADRS scores during the respective phase, and presence of comorbid conditions affecting sexual function, as fixed effects, and recruitment site and individual as random effects variables. ESC+ARI analysis was additionally adjusted for ARI dosage.

|  | ***Phase I*** | | | | | | ***Phase II: ESC-Only*** | | | | | | ***Phase II: ESC+ARI*** | | | | | |
| --- | --- | --- | --- | --- | --- | --- | --- | --- | --- | --- | --- | --- | --- | --- | --- | --- | --- | --- |
| **Fixed Effects** | **Estimates** | **SE** | **CI (95%)** | **P-Value** | **df** | **Estimates** | | **SE** | **CI (95%)** | **P-Value** | **df** | **Estimates** | | **SE** | **CI (95%)** | **P-Value** | **df** |  |
| **Intercept** | 10.72 | 1.62 | 7.51 – 13.93 | **<0.001**  ******* | 145 | 14.47 | | 2.25 | 9.97 – 18.97 | **<0.001**  ******* | 56 | 17.93 | | 3.61 | 10.72 – 25.13 | **<0.001**  ******* | 64 |  |
| **Week** | -0.05 | 0.17 | -0.38 – 0.28 | 0.760 | 139 | -0.23 | | 0.10 | -0.44 – -0.03 | **0.025**  ***** | 54 | -0.30 | | 0.15 | -0.61 – 0.01 | 0.058 | 64 |  |
| **CYP2C19 IM+PM** | 1.11 | 0.61 | -0.11 – 2.32 | 0.074 | 145 | -3.62 | | 1.61 | -6.84 – -0.39 | **0.029*** | 56 | 0.22 | | 1.61 | -3.01 – 3.45 | 0.891 | 53 |  |
| **CYP2C19 RM+UM** | -0.00 | 0.60 | -1.19 – 1.19 | 0.997 | 145 | -1.51 | | 1.55 | -4.61 – 1.59 | 0.332 | 56 | -1.75 | | 1.53 | -4.82 – 1.32 | 0.259 | 53 |  |
| **CYP2D6 IM+PM** | 0.45 | 0.51 | -0.56 – 1.47 | 0.381 | 145 | -3.01 | | 1.28 | -5.57 – -0.45 | **0.022**  ***** | 56 | -0.33 | | 1.41 | -3.16 – 2.50 | 0.816 | 53 |  |
| **CYP2C19 IM+PM*Week** | -0.06 | 0.08 | -0.22 – 0.10 | 0.432 | 139 | 0.44 | | 0.11 | 0.21 – 0.67 | **<0.001**  ******* | 54 | 0.12 | | 0.12 | -0.12 – 0.35 | 0.321 | 64 |  |
| **CYP2C19 RM+UM*Week** | -0.03 | 0.08 | -0.19 – 0.12 | 0.669 | 139 | 0.13 | | 0.11 | -0.09 – 0.34 | 0.255 | 54 | 0.10 | | 0.11 | -0.12 – 0.33 | 0.359 | 64 |  |
| **CYP2D6 IM+PM*Week** | -0.16 | 0.07 | -0.29 – -0.02 | **0.026**  ***** | 139 | 0.13 | | 0.09 | -0.05 – 0.31 | 0.156 | 54 | 0.01 | | 0.10 | -0.20 – 0.21 | 0.940 | 64 |  |
| **Random Effects** | | | | | | | | | | | | | | | | | | |
| N | 158 _SUBJLABEL_ | | | | | | 69 _SUBJLABEL_ | | | | | | 71 _SUBJLABEL_ | | | | | |
|  | 6 _SITESYMBOL_ | | | | | | 6 _SITESYMBOL_ | | | | | | 6 _SITESYMBOL_ | | | | | |
| Observations | 303 | | | | | | 129 | | | | | | 141 | | | | | |

ARI = Aripiprazole; CI = confidence interval; ESC = Escitalopram;

IM = Intermediate Metabolizer; NM = Normal Metabolizer; PM = Poor Metabolizer; RM = Rapid Metabolizer; SE = standard error; UM = Ultra-rapid Metabolizer.

**p*<0.05   ** *p*<0.01   *** *p*<0.001

**Figure S2.** Interaction between all three genes, *CYP2C19, CYP2D6,* or *ABCB1,* on treatment-related changes in SF and SS over time.

**
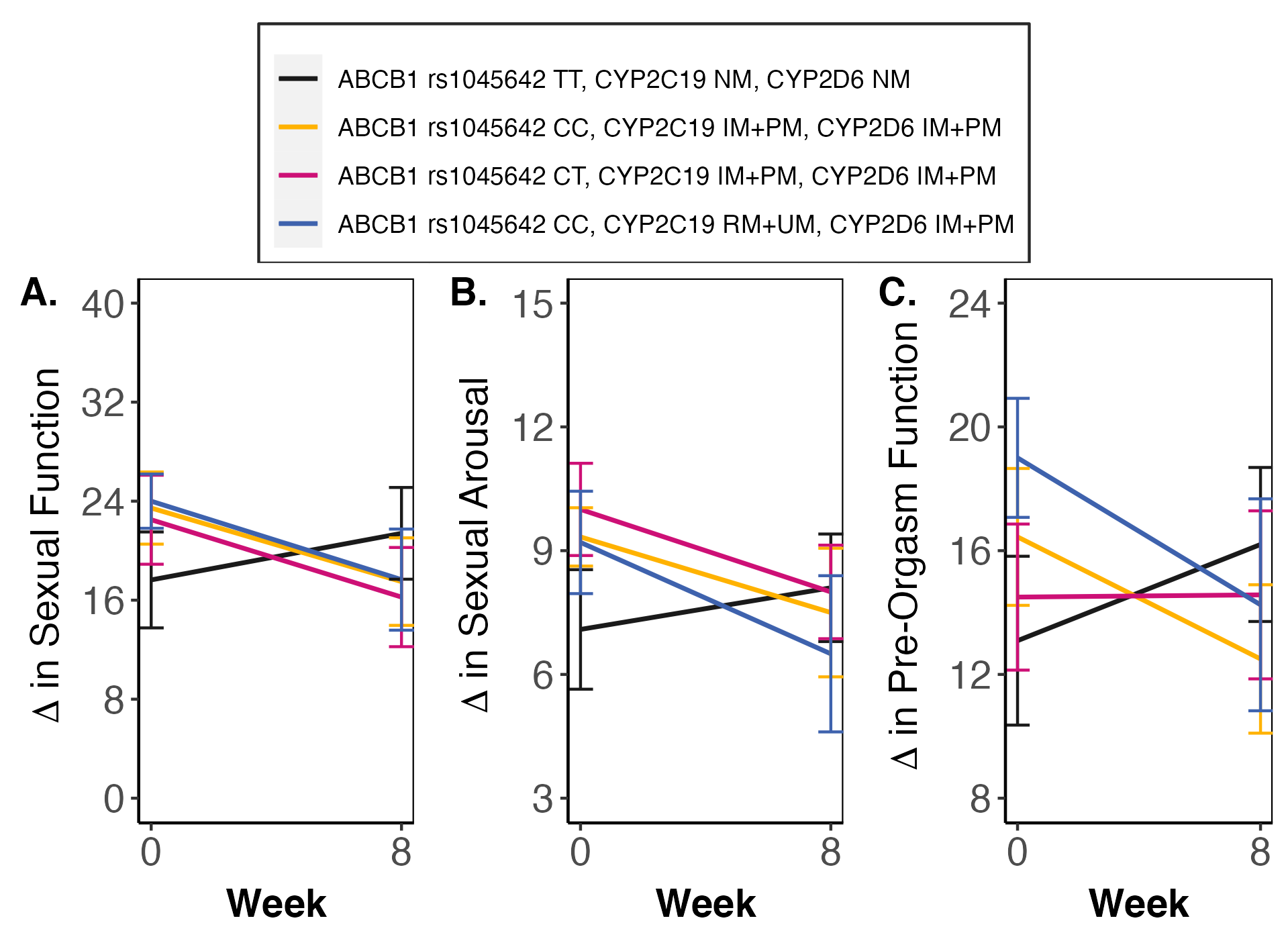
**

**(A)** Significantly greater mean decline in SF from baseline were observed in *CYP2D6* and *CYP2C19* IM+PMs who had the CC (B=-4.37, 95% CI: [-7.09, -1.63]) or CT (B=-2.65, 95% CI: [-4.94, -0.36]) genotype at the *ABCB1* rs1045642 SNP or were *CYP2D6* IM+PM, *CYP2C19* RM+UM and had the rs1045642 CC genotype (B=-5.54, 95% CI: [-8.25, -2.82]) compared to those with normal CYP function and P-gp expression (i.e., NMs of *CYP2D6* and *CYP2C19* with the rs1045642 TT genotype). Likewise, the same effect was observed for the **(B)** arousal and **(C)** pre-orgasm subdomains of SF.

Error bars are standard error.

ESC = Escitalopram;

IM = Intermediate Metabolizer; NM = Normal Metabolizer; PM = Poor Metabolizer; RM = Rapid Metabolizer; SF = Sexual Function; UM = Ultra-rapid Metabolizer.

**Table S6**. Spearman correlations of measures of serum concentrations with changes (Δ) in sexual function, its domains, and sexual satisfaction at Week 8 and 16.

|  | **Week 2 of Phase I** | | |
| --- | --- | --- | --- |
|  | **[ESC]** | **[S-DCT]** | **[S-DCT]/[ESC]** |
| **Δ Sexual function** | -0.16 | 0.042 | 0.18 |
| **Δ Arousal** | -0.091 | 0.035 | 0.12 |
| **Δ Desire** | -0.15 | -0.014 | 0.14 |
| **Δ Orgasm** | -0.083 | 0.14 | 0.17 |
| **Δ Pre-Orgasm** | -0.13 | 0.026 | 0.14 |
| **Δ Sexual Satisfaction** | 0.0047 | -0.12 | -0.077 |

|  | **ESC-Only: Week 16 of Phase II** | | |
| --- | --- | --- | --- |
|  | **[ESC]** | **[S-DCT]** | **[S-DCT]/[ESC]** |
| **Δ Sexual function** | -0.055 | -0.25 | -0.068 |
| **Δ Arousal** | -0.1 | -0.27 | -0.047 |
| **Δ Desire** | -0.035 | -0.11 | -0.016 |
| **Δ Orgasm** | -0.12 | -0.24 | 0.046 |
| **Δ Pre-Orgasm** | -0.064 | -0.22 | -0.038 |
| **Δ Sexual Satisfaction** | 0.15 | -0.24 | -**0.33*** |

|  | **ESC+ARI: Week 16 of Phase II** | | | | | |
| --- | --- | --- | --- | --- | --- | --- |
|  | **[ESC]** | **[S-DCT]** | **[S-DCT]/[ESC]** | **[ARI]** | **[DHA]** | **[DHA]/[ARI]** |
| **Δ Sexual function** | 0.16 | 0.24 | 0.064 | 0.15 | 0.073 | -0.14 |
| **Δ Arousal** | 0.21 | 0.12 | -0.095 | 0.084 | 0.063 | -0.077 |
| **Δ Desire** | 0.16 | 0.23 | 0.063 | 0.12 | 0.026 | -0.13 |
| **Δ Orgasm** | -0.014 | 0.098 | 0.072 | 0.097 | 0.044 | -0.12 |
| **Δ Pre-Orgasm** | 0.22 | 0.21 | -0.013 | 0.13 | 0.049 | -0.12 |
| **Δ Sexual Satisfaction** | -0.09 | 0.022 | 0.23 | -0.06 | 0.041 | 0.19 |

ARI = Aripiprazole; DHA = dehydroaripiprazole; ESC = Escitalopram; MADRS = Montgomery-Asberg Depression Rating Scale; S-DCT = S-desmethylcitalopram.

**q* < 0.05

**Figure S3.** Dose-adjusted ESC concentrations in serum for Phase I and II by *CYP2C19* and *CYP2D6* metabolizer groups.


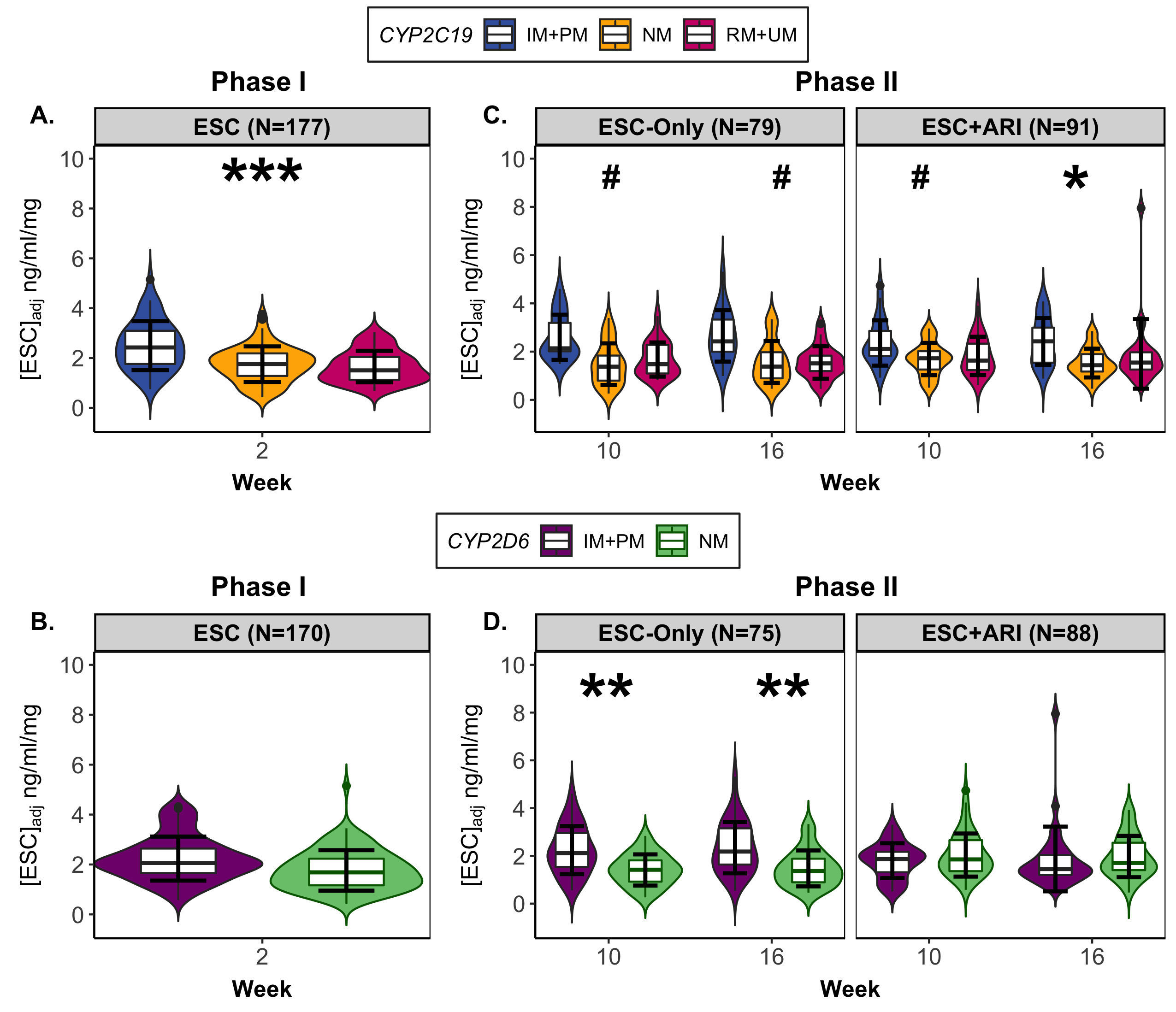


During Phase I, (**A**) *CYP2C19* IM+PMs showed higher mean ESC_adj_ concentrations relative to NMs, whereas there was no significant difference in ESC_adj_ concentrations between NMs and RM+UMs. (**B**) A significant difference in ESC_adj_ concentrations was not observed between *CYP2D6* metabolizer group. During Phase II, for the ESC-Only treatment arm, (**C**) *CYP2C19* and (**D**) *CYP2D6* IM+PMs compared to NMs had higher ESC levels in serum. In the ESC+ARI treatment arm, ESC_adj_ serum levels were associated with only (**C**) *CYP2C19*, but not (**D**) *CYP2D6*, with higher ESC_adj_ concentrations in *CYP2C19* IM+PMs relative to NMs.

All linear regression analyses were adjusted for age, ancestry, sex, site, time since last dose, *CYP2C19* and *CYP2D6* metabolizer groups. Error bars represent standard error.

ARI = Aripiprazole; ESC = Escitalopram;

IM = Intermediate Metabolizer; NM = Normal Metabolizer; PM = Poor Metabolizer; RM = Rapid Metabolizer; UM = Ultra-rapid Metabolizer.

* *q*<0.05; ** *q*<0.01; *** *q*<0.001; # indicates trend with *q* between 0.050 to 0.070.

**Figure S4.** Dose-adjusted serum S-DCT/ESC_adj_ ratio for Phase I and II by *CYP2C19* and *CYP2D6* metabolizer groups.


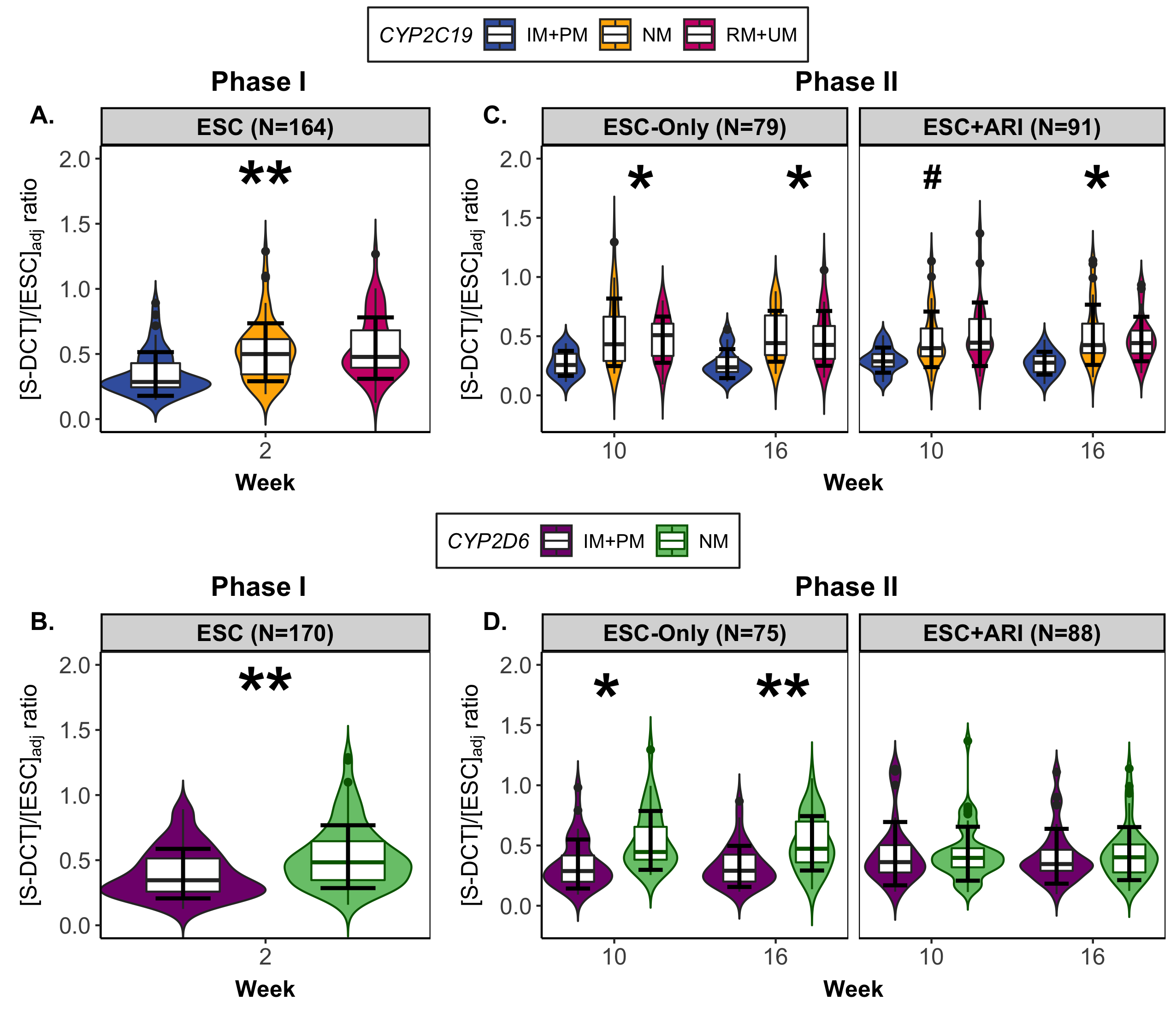


During Phase I, (**A**) *CYP2C19* and (**B**) *CYP2D6* IM+PMs showed lower mean S-DCT_ad_/ESC_adj_ ratio relative to NMs. Likewise, during Phase II, in the ESC-Only and ESC+ARI treatment arms, (**C**) *CYP2C19* IM+PMs compared to NMs had lower mean S-DCT_ad_/ESC_adj_ ratio in serum. (**D**) For *CYP2D6*, IM+PMs displayed lower S-DCT_ad_/ESC_adj_ ratio in ESC-Only, whereas S-DCT_ad_/ESC_adj_ ratio was not associated with *CYP2D6* metabolizer group in the ESC+ARI treatment arm.

All linear regression analyses were adjusted for age, ancestry, sex, site, time since last dose, *CYP2C19* and *CYP2D6* metabolizer groups. Error bars represent standard error.

ARI = Aripiprazole; ESC = Escitalopram;

IM = Intermediate Metabolizer; NM = Normal Metabolizer; PM = Poor Metabolizer; RM = Rapid Metabolizer; UM = Ultra-rapid Metabolizer.

* *q*<0.05; ** *q*<0.01; *** *q*<0.001.
